# Evaluation of Clinically Meaningful Changes in Measures of Frailty

**DOI:** 10.1093/gerona/glaa003

**Published:** 2020-03-07

**Authors:** Il-Young Jang, Hee-Won Jung, Hea Yon Lee, Hyungchul Park, Eunju Lee, Dae Hyun Kim

**Affiliations:** 1 Department of Internal Medicine, Asan Medical Center, University of Ulsan College of Medicine, Seoul, Republic of Korea; 2 PyeongChang Health Center and County Hospital, PyeongChang, Gangwon-Do, Republic of Korea; 3 Department of Internal Medicine, Seoul National University Hospital, Seoul, Republic of Korea; 4 Marcus Institute for Aging Research, Hebrew SeniorLife, Boston, MA; 5 Division of Gerontology, Department of Medicine, Beth Israel Deaconess Medical Center, Boston, MA

**Keywords:** Frailty, Clinically meaningful change, Responsiveness

## Abstract

**Background:**

To determine the clinically meaningful changes and responsiveness of widely used frailty measures.

**Methods:**

We analyzed data from a prospective cohort study of 1,135 community-dwelling older adults who underwent assessments of frailty and health-related quality of life using the EuroQol-5D at baseline and 1 year later. Frailty measures included deficit-accumulation frailty index (FI); frailty phenotype; Fatigue, Resistance, Ambulation, Illness, and Loss of Weight scale; and the Study of Osteoporotic Fracture (SOF) index. We determined the clinically meaningful changes by the distribution-based method and the anchor-based method using the EuroQol-5D score and responsiveness indices.

**Results:**

Frailty measures were available in 925 participants at 1 year (81.5%). Based on the distribution-based method, small and large clinically meaningful changes were 0.019 and 0.057 for FI, 0.249 and 0.623 for frailty phenotype, 0.235 and 0.587 for FRAIL scale, and 0.116 and 0.289 for SOF index, respectively. The anchor-based estimates of small and large changes were 0.028 and 0.076 for FI, 0.097 and 0.607 for frailty phenotype, 0.269 and 0.368 for FRAIL scale, and 0.023 and 0.287 for SOF index, respectively. Based on the responsiveness index, per-group sample sizes to achieve 80% power in clinical trials, ranged from 51 (FI) to 7,272 (SOF index) for a small change and 9 (FI) to 133 (FRAIL scale) for a large change.

**Conclusions:**

The estimates of clinically meaningful change of frailty measures can inform the choice of frailty measures to track longitudinal changes of frailty in clinical trials and clinical care of community-dwelling older adults.

To date, frailty measures have been mainly used for risk prediction of future adverse health outcomes in older adults (1). As more research targets frailty as an outcome, understanding the responsiveness of frailty measures and clinically meaningful changes can inform the choice of frailty measures and calculate the sample size for a clinical trial. In addition, it can provide useful means to monitor the response to a treatment in clinical care.

How likely a frailty measure changes longitudinally with deterioration of health status has not been well investigated. The frailty phenotype measures unintentional weight loss, exhaustion, inactivity, slow gait, and weak grip strength (2), all of which can improve or worsen with health status. The deficit-accumulation frailty index (FI), which quantifies frailty as a proportion of abnormalities from a list of age-related health deficits (3–5), is increasingly applied to existing databases, including administrative claims data (6,7) and electronic health records (8,9). A typical FI measures the presence or absence of chronic diseases, however it may not capture worsening of the existing conditions. The longitudinal changes of other simple frailty measures, such as the Fatigue, Resistance, Ambulation, Illness, and Loss of weight (FRAIL) scale (10,11) or the Study of Osteoporotic Fracture (SOF) index (12), are not well known. Apart from FI, which gives a continuous score ranging from 0 to 1, the other measures give discrete scores from 0 to 3 (SOF index) or 5 (frailty phenotype and FRAIL scale). What constitutes a clinically meaningful change for each measure remains uncertain.

The objective of this study was to determine the clinically meaningful changes for four commonly used frailty measures—frailty phenotype, FI, FRAIL scale, and SOF index—more than 1 year among community-dwelling older adults. We applied the distribution-based method and the anchor-based method (13–16) to define the changes in each frailty measures that corresponded to small and large clinical effects. Based on these estimates, we determined a sample size of a clinical trial to detect small and large effect sizes.

## Method

### Study Population

The Aging Study of PyeongChang Rural Area (ASPRA) study is a population-based, prospective cohort study to investigate frailty and geriatric syndromes in community-dwelling older adults (17). The Institutional Review Board of Asan Medical Center, Seoul, Korea, approved the study and participants provided informed consent. Enrollment took place between October 2014 and December 2018 in PyeongChang County, located 180 km east of Seoul, Korea. Eligible participants were those who were (i) 65 years or older; (ii) registered in the National Healthcare Service (all Korean citizens are registered in the system); (iii) ambulatory with or without an assistive device; and (iv) living at home. Excluded were those who were living in a nursing home, hospitalized, or receiving nursing home-level care at home at the time of enrollment. More than 90% of the eligible people were enrolled and their sociodemographic characteristics were similar to people residing in rural areas from a national survey of Korean people, except for higher proportions of ASPRA participants engaged in agriculture (53.4% vs 25.1%) and with low education (44.8% vs 22.6%) (17). For this study, we analyzed data collected at baseline and 1-year follow-up in 1,135 people with baseline frailty and health-related quality-of-life assessments in the ASPRA population.

### Frailty Assessment

We assessed frailty at baseline and 1-year follow-up using the following measures of frailty.

1) Deficit-accumulation FI: According to the method proposed by Rockwood et al. (3–5), we calculated the proportion of 43 age-related health deficits (range: 0–1). Of these deficit items, 38 items were self-reported and 5 items were derived from performance tests (see the complete list of items in Supplementary Table 1). We calculated two versions of FI: a FI based on all 43 items and another FI based on 38 self-reported items only.
2) Frailty phenotype: Frailty was defined based on unintentional weight loss, self-reported exhaustion, physical inactivity, slow gait, and weak grip strength (2). How these assessments were performed in our study has been previously described (17). Although it classifies people into robust (0 positive items), prefrail (1–2 positive items), or frail states (≥3 positive items), we used it as a continuous scale (range: 0–5).
3) FRAIL scale: The FRAIL scale consists of five self-reported fatigue, resistance (difficulty walking up 10 steps without resting), ambulation (difficulty walking 300 m), illnesses (≥5 chronic conditions) and loss of weight (>5% in the past year) (10). We used the Korean version of FRAIL scale that has been validated (18). Analogous to the frailty phenotype, we used the continuous scale (range: 0–5).
4) SOF index: This index consists of unintentional weight loss, inability to rise from a chair five times without use of arms, and reduced energy level (12). Although it classifies people into robust (0 positive items), prefrail (1 positive item), or frail states (≥2 positive items), we used the continuous scale (range: 0–3).

### Assessment of Health-Related Quality of Life as an Anchor Measure of Health Status

Health-related quality of life was assessed at baseline and 1 year using the EuroQol-5D (EQ-5D-3L descriptive system), which is a standardized measure of health status for clinical and economic appraisal (19,20). Each of the five dimensions (mobility, self-care, usual activities, pain or discomfort, and anxiety or depression) were assessed in three levels of having no, some, or extreme problems. The EQ-5D was used as an anchor to derive clinically meaningful changes as described later (see Statistical Analysis). We classified participants into three categories based on the EQ-5D change more than 1 year (follow-up score minus baseline score): no decline (>−0.1), small decline (−0.1 to −0.2), and large decline (<−0.2).

### Statistical Analysis

The analysis was performed in SPSS version 21.0 (IBM Corporation, Armonk, NY) and R software version 3.3.3 (R Foundation for Statistical Computing, Vienna, Austria). A two-sided *P*-value < 0.05 was considered statistically significant. We summarized the distribution of each frailty measure at baseline using a mean with standard deviation (*SD*).

To estimate the clinically meaningful changes, we employed both distribution-based and anchor-based methods (14–16). When the distribution-based method was used, we defined the small and large clinically meaningful changes as 0.2 and 0.5 times the *SD* of a frailty measure at baseline, respectively (21,22). When the anchor-based method was used, the small clinically meaningful change was calculated as a difference in the mean change of a frailty measure (follow-up score minus baseline score) between those with a small EQ-5D decline and those with no decline. Similarly, the large clinically meaningful change was calculated by contrasting those with a large EQ-5D decline and those with no decline.

We also estimated the responsiveness index (RI) (23) for each frailty measure, which is a ratio of clinically meaningful change (estimated from the anchor-based method) to the *SD* of individual change scores in those with no EQ-5D decline. Clinical trials that adopt a frailty measure with high RI require a smaller sample size to detect the clinically meaningful change. We estimated the sample size for a 1:1 parallel-arm clinical trial to detect small and large clinically meaningful changes more than 1 year, with a Type I error of 5% and 80% power.

## Results

The mean age of the study population was 73.8 years and 53.1% was female (Table 1). The mean (*SD*) frailty score at baseline was 0.11 (0.10) for the 38-item FI, 0.14 (0.11) for the 43-item FI, 1.46 (1.25) for frailty phenotype, 1.13 (1.17) for FRAIL scale, and 0.33 (0.58) for SOF index. These mean scores were consistent in the prefrail state. After 1 year, 925 (81.5%) completed the assessment (1.4% died, 6.5% were institutionalized, 8.7% did not respond to the follow-up assessment, and 1.9% were lost to follow-up).

**Table 1.** Characteristics of Study Population (*N* = 1,135)

Characteristics	Mean (*SD*) or *N* (%)
Age, years	73.8 (6.5)
Female	603 (53.1)
FI (38 items) (range: 0–1)^a^	0.11 (0.10)
FI (43 items) (range: 0–1)^b^	0.14 (0.11)
Frailty phenotype (range: 0–5)	1.46 (1.25)
FRAIL scale (range: 0–5)	1.13 (1.17)
SOF index (range: 0–3)	0.33 (0.58)
Follow-up status at 1 year	
Died	16 (1.4)
Institutionalized	74 (6.5)
Noninstitutionalized with no follow-up data	99 (8.7)
Noninstitutionalized with follow-up data	925 (81.5)
Vital status unknown	21 (1.9)
EuroQol-5D at baseline	0.83 (0.15)
EuroQol-5D at 1-year follow-up (*N* = 925)	0.83 (0.13)
No decline	751 (81.2)
Small decline	135 (14.6)
Large decline	39 (4.2)

*Note*: FI = frailty index; FRAIL = Fatigue, Resistance, Ambulation, Illness, and Loss of weight; SOF = Study of Osteoporotic Fracture; *SD* = standard deviation.

^a^The 38-item FI was calculated from 38 self-reported items.

^b^The 43-item FI was calculated from 38 self-reported items and 5 performance test items.

**Figure 1.**
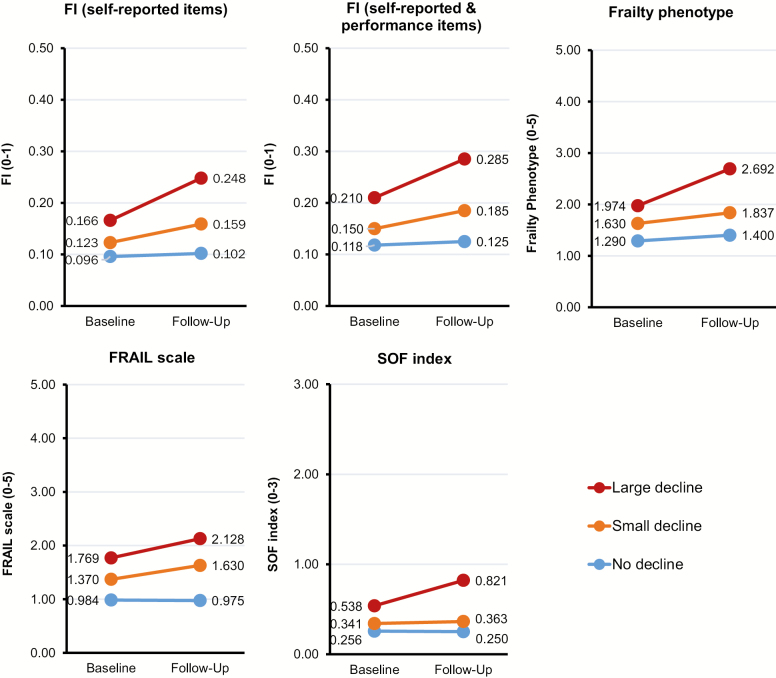
One-year changes in frailty measures by EQ-5D change. The 38-item FI was calculated from 38 self-reported items. The 43-item FI was calculated from 38 self-reported items and 5 performance test items. The mean scores of individual frailty measures at baseline and 1-year follow-up assessments were plotted. *Note*: FI = frailty index; FRAIL, Fatigue, Resistance, Ambulation, Illness, and Loss of weight; SOF = Study of Osteoporotic Fracture.

Of those with follow-up data, 135 (14.6%) had a small decline and 39 (4.2%) had a large decline in EQ-5D. All frailty measures changed little in participants with no decline in EQ-5D, whereas their increment was greater in those with a large EQ-5D decline (Figure 1 and Supplementary Table 2).

### Clinically Meaningful Changes in Frailty Measures

The estimates of small and large clinically meaningful changes are given in Table 2. When the distribution-based method was used, small clinically meaningful changes were 0.019 for the 38-item FI, 0.023 for the 43-item FI, 0.249 for frailty phenotype, 0.235 for FRAIL scale, and 0.116 for SOF index. Large clinically meaningful changes were 0.049, 0.057, 0.623, 0.587, and 0.289, respectively. The anchor-based estimates of small and large changes for the 38-item FI (0.030 and 0.076, respectively) and 43-item FI (0.028 and 0.067, respectively) were similar to the distribution-based estimates. However, the two methods resulted in different estimates for the frailty phenotype (small change: 0.249 from distribution-based method vs 0.097 from anchor-based method), FRAIL scale (large change: 0.587 from distribution-based method vs 0.368 from anchor-based method), and SOF index (small change: 0.116 from distribution-based method vs 0.023 from anchor-based method).

**Table 2.** One-Year Clinically Meaningful Changes in Frailty Measures

Frailty Measures	Distribution-Based		Anchor- Based	
	Small	Large	Small	Large
FI (38 items) (range: 0–1)*	0.019	0.049	0.030	0.076
FI (43 items) (range: 0–1)^†^	0.023	0.057	0.028	0.067
Frailty phenotype (range: 0–5)	0.249	0.623	0.097	0.607
FRAIL scale (range: 0–5)	0.235	0.587	0.269	0.368
SOF index (range: 0–3)	0.116	0.289	0.023	0.287

*Note*: FI = frailty index; FRAIL = Fatigue, Resistance, Ambulation, Illness, and Loss of weight; SOF = Study of Osteoporotic Fracture; SD = standard deviation.

*The 38-item FI was calculated from 38 self-reported items.

^†^The 43-item FI was calculated from 38 self-reported items and 5 performance test items.

### Responsiveness of Frailty Measures and Sample Size Considerations for Clinical Trials

The FIs showed higher RIs than other frailty measures (Table 3). The RI for detecting a small clinically meaningful change was 0.552 for the 38-item FI, 0.417 for the 43-item FI, 0.083 for frailty phenotype, 0.258 for FRAIL scale, and 0.046 for SOF index. The RI for a large clinically meaningful change was 1.319, 1.154, 0.502, 0.343, and 0.471, respectively. With a Type I error of 5% and 80% power, a 1:1 parallel-arm clinical trial requires 9 (38-item FI) to 133 (FRAIL scale) participants per group to detect a large change in a frailty measure over 1 year. To detect a small change over 1 year, the sample size per group ranges from 51 (38-item FI) to 7,272 (SOF index) participants.

**Table 3.** Responsiveness of Frailty Measures and Sample Size Considerations for Clinical Trials

Frailty Measures	Clinically Meaningful Change*	*SD* of Change in Stable Group	Responsiveness Index	Sample Size (Per Group)^†^
FI (38 items)^‡^				
Small change	0.030	0.055	0.552	51
Large change	0.076	0.058	1.319	9
FI (43 items)^§^				
Small change	0.028	0.068	0.417	90
Large change	0.067	0.058	1.154	12
Frailty phenotype				
Small change	0.097	1.172	0.083	2,294
Large change	0.607	1.210	0.502	62
FRAIL scale				
Small change	0.269	1.041	0.258	236
Large change	0.368	1.074	0.343	133
SOF index				
Small change	0.028	0.593	0.046	7,272
Large change	0.287	0.610	0.471	71

*Note*: FI = frailty index; FRAIL = Fatigue, Resistance, Ambulation, Illness, and Loss of weight; SOF = Study of Osteoporotic Fracture; *SD* = standard deviation.

*Clinically meaningful changes were estimated from the anchor-based method.

^†^Sample size was estimated for a 1:1 two-arm clinical trial to detect small and large clinically meaningful changes, with 5% Type I error and 80% power.

^‡^The 38-item FI was calculated from 38 self-reported items.

^§^The 43-item FI was calculated from 38 self-reported items and 5 performance test items.

## Discussion

By applying distribution-based and anchor-based methods, we estimated small and large clinically meaningful changes in the widely used frailty measures in community-dwelling older adults. Our results suggest that FIs are more responsive than other frailty measures to the change in health status measured using EQ-5D. This information is useful for choosing a frailty measure and evaluating the clinical significance of an intervention effect in clinical trials. In clinical practice, it may facilitate clinical interpretation of different frailty measures to inform prognostication and monitor health status over time in older adults.

Frailty is a core measure of health status and strong predictor for adverse health outcomes in older adults, yet its use as an outcome measure in clinical trials of geriatric populations remains controversial (24–26). Other than feasibility of measurement, barriers to adopt frailty measures include lack of information on clinically meaningful differences and responsiveness of frailty measures. Our study provides this key information based on the population distribution of frailty measures (distribution-based method) and comparison of changes in frailty measures between individuals who experienced health decline and those who did not according to EQ5D (anchor-based method). These methods resulted in similar estimates of clinically meaningful difference for the full FI or self-reported item-only FI, but inconsistent estimates for frailty phenotype, FRAIL scale, and SOF index. Although the estimates from the anchor-based method may depend on the choice of an anchor, EQ5D is a well-established measure of health status assessed in five domains (19,20). Therefore, we believe that the anchor-based method is more likely to provide estimates of clinical significance than the distribution-based method.

Our results revealed a large variation of responsiveness of frailty measures, with RIs ranging from 0.046 (SOF index) to 0.552 (FI) for a small clinically meaningful change and from 0.343 (FRAIL scale) to 1.319 (FI) for a large clinically meaningful change. The sample size per group in a clinical trial was 51 (FI) to 7,272 (SOF index) for a small change and 9 (FI) to 133 (FRAIL scale) for a large change. In the Lifestyle Interventions and Independence for Elders (LIFE) trial (*n* = 1,635), a structured physical activity program compared with health education failed to show a difference in frailty measured by SOF index (27). Given the low responsiveness of SOF index observed in our study, choice of an alternative frailty measure (eg, FI) may have resulted in a different conclusion.

The strengths of our study include enrollment of more than 90% of eligible individuals residing in the study areas; standardized assessments of several frailty measures and health-related quality of life; use of alternative approaches to estimate clinically meaningful differences; and repeated measurements of frailty with a high retention rate at 1 year. A major limitation of our study is that our estimates may depend on the distribution of frailty and EQ5D, and rate of EQ5D decline in older Koreans in rural areas. Generalizability of our results to western populations or older adults living in urban areas is unclear. As mentioned earlier, use of a different anchor may result in different estimates. Another limitation is that we only examined clinically meaningful changes of frailty measures in association with health decline with aging in the absence of any specific intervention. How these frailty measures respond to an intervention targeted to improve frailty (eg, exercise and nutrition) (28,29) remains to be determined. Despite these limitations, our results fill in the important gap in geriatric research. Future research should replicate our analysis in independent populations, in intervention studies, and by using alternative anchors of health status.

In summary, we estimated small and large clinically meaningful changes and responsiveness of widely used frailty measures for community-dwelling older adults. We hope that this information can inform the choice of frailty measures to track longitudinal changes in frailty in clinical trials and clinical care.

## Supplementary Material

glaa003_suppl_Supplementary_Materials
