# Supplementary material for: Evaluation of Clinically Meaningful Changes in Measures of Frailty
Source: J Gerontol A Biol Sci Med Sci. 2020 Mar 7;75(6):1143–7. doi: 10.1093/gerona/glaa003 (PMC7243580; doi:10.1093/gerona/glaa003)
Supplement: glaa003_suppl_Supplementary_Materials [file glaa003_suppl_supplementary_materials.docx]

**SUPPLEMENTARY MATERIALS**

**Evaluation of Clinically Meaningful Changes in Measures of Frailty**

Il-Young Jang, Hee-Won Jung, Hea Yon Lee, Hyungchul Park, Eunju Lee,

and Dae Hyun Kim

**TABLE OF CONTENTS**

| **Supplementary Table 1.** | Items for Deficit-Accumulation Frailty Index |
| --- | --- |
| **Supplementary Table 2.** | One-Year Changes in Frailty Measures By EQ-5D Decline |

**Supplementary Table 1. Items for Deficit-Accumulation Frailty Index**

| **Self-reported items (38 items)** |  |
| --- | --- |
| • Hypertension  • Diabetes  • Cancer (other than a minor skin cancer),  • Chronic lung disease  • Heart attack  • Congestive heart failure  • Angina  • Asthma  • Arthritis  • Stroke  • Kidney disease  • Dementia  • Falls  • Depression  • Anxiety disorder  • Bathing  • Continence  • Dressing  • Eating | • Using telephone  • Toileting  • Transferring  • Washing face and hands  • Food preparation  • Doing household chores  • Going out short distance  • Grooming  • Handling finances  • Laundry  • Managing own medications  • Shopping  • Transportation  • Fatigue  • Resistance  • Ambulation  • Low physical activity  • At risk of malnutrition  • Polypharmacy |
| **Performance test items (5 items)** |  |
| • Grip strength  • Balance  • Chair stand | • Gait speed  • Mini-Mental Status Examination |

**Supplementary Table 2. One-Year Changes in Frailty Measures By EQ-5D Decline**

| **Frailty Measure** | **No decline**  **(N=751)** | **Small decline (N=135)** | **Large decline (N=39)** | **P-value** |
| --- | --- | --- | --- | --- |
| **FI (38 items)** |  |  |  |  |
| Baseline, mean (SD) | 0.096 (0.079) | 0.123 (0.074) | 0.166 (0.098) |  |
| Follow-up, mean (SD) | 0.102 (0.081) | 0.159 (0.085) | 0.248 (0.112) |  |
| Change (95% CI) | 0.006 (0.002, 0.010) | 0.04 (0.024, 0.047) | 0.082 (0.057, 0.107) | <0.001 |
| **FI (43 items)** |  |  |  |  |
| Baseline, mean (SD) | 0.118 (0.094) | 0.150 (0.090) | 0.210 (0.118) |  |
| Follow-up, mean (SD) | 0.125 (0.095) | 0.185 (0.099) | 0.285 (0.128) |  |
| Change (95% CI) | 0.007 (0.003, 0.011) | 0.035 (0.024, 0.047) | 0.075 (0.050, 0.992) | <0.001 |
| **Frailty phenotype** |  |  |  |  |
| Baseline, mean (SD) | 1.290 (1.169) | 1.630 (1.297) | 1.974 (1.246) |  |
| Follow-up, mean (SD) | 1.400 (1.041) | 1.837 (1.121) | 2.692 (1.173) |  |
| Change (95% CI) | 0.111 (0.027, 0.194) | 0.207 (-0.032, 0.446) | 0.718 (0.298, 1.138) | 0.008 |
| **FRAIL scale** |  |  |  |  |
| Baseline, mean (SD) | 0.984 (1.115) | 1.370 (1.138) | 1.769 (1.202) |  |
| Follow-up, mean (SD) | 0.975 (1.160) | 1.630 (1.138) | 2.128 (1.196) |  |
| Change (95% CI) | -0.009 (-0.084, 0.065) | 0.259 (0.051, 0.467) | 0.359 (-0.024, 0.742) | 0.005 |
| **SOF index** |  |  |  |  |
| Baseline, mean (SD) | 0.256 (0.486) | 0.341 (0.562) | 0.538 (0.682) |  |
| Follow-up, mean (SD) | 0.250 (0.507) | 0.363 (0.594) | 0.821 (0.756) |  |
| Change (95% CI) | -0.005 (-0.048, 0.037) | 0.022 (-0.096, 0.141) | 0.282 (0.004, 0.560) | 0.019 |

Abbreviations: FI, frailty index; FRAIL, Fatigue, Resistance, Ambulation, Illness, and Loss of weight; SOF, Study of Osteoporotic Fracture; SD, standard deviation.

^a^The 38-item FI was calculated from 38 self-reported items.

^b^The 43-item FI was calculated from 38 self-reported items and 5 performance test items.
